# Medical Society of Kentucky

**Published:** 1855-12

**Authors:** 


### MEDICAL SOCIETY OF KENTUCKY.

OUR Kentucky readers will bear in mind the meeting of the State Medical Society, which is to be held in Frankfort on the first Monday in February. We hope to see a large meeting.

